# Surgical Treatment of Dysphagia Lusoria Caused by Right-Aortic Arch with Kommerell Diverticulum: Left Heart Bypass without Subclavian Revascularization

**DOI:** 10.1055/s-0038-1639346

**Published:** 2018-07-27

**Authors:** Mariano Camporrotondo, Paz Ricapito, Juan Carlos Espinoza, Fernando Piccinini, Mariano Vrancic, Gustavo Avegliano, Daniel Navia

**Affiliations:** 1 Cardiac Surgery Department, Instituto Cardiovascular de Buenos Aires, Buenos Aires, Argentina

**Keywords:** right aortic arch, dysphagia lusoria, Kommerell diverticulum

## Abstract

The authors present the case of a 26-year-old patient suffering from dysphagia because of compression by a Kommerell diverticulum in right aortic arch anomaly. Open surgical arch and descending aorta replacement with left heart bypass without left subclavian artery reimplantation was performed.

## Introduction

Right-sided aortic arch and aberrant left subclavian artery (LSA) might cause symptoms due to the compression of the esophagus related to aneurysmal dilatation of the aberrant subclavian. Endovascular treatment is not indicated due to mechanical symptoms. Open surgical repair is a challenge and it requires careful planning.

## Case Presentation

**Fig. 1:**
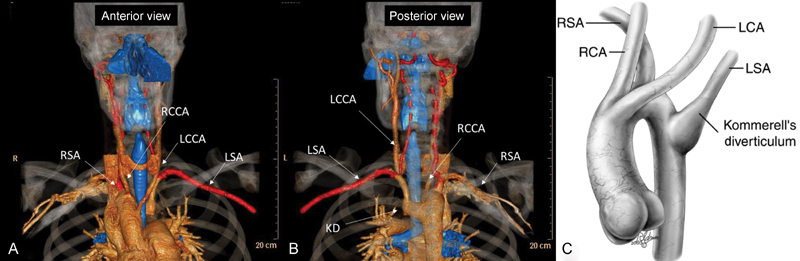
Preoperative computed tomography angiography of the right aortic arch with aberrant origin of LSA. ( **A** ) Anterior view with the aortic arch crosses over the right bronchus (blue), the definition of a right aortic arch. The order of origin of the brachiocephalic arteries arising from the aorta is LCCA, RCCA, RSA, and LSA. ( **B** ) Posterior view with an outpouching at the base of the LSA that is termed a KD. ( **C** ) Drawing of a right aortic arch with an aberrant origin of the LSA. (Reprinted with permission from Backer CL, Mavroudis C, Rigsby CK, Holinger LD. Trends in vascular ring surgery. J Thorac Cardiovasc Surg 2005;129:1339–1347, Elsevier.) KD, Kommerell's diverticulum; LCA, left common artery; LCCA, left common carotid artery; LSA, left subclavian artery; RCA, right common artery; RCCA, right common carotid artery; RSA, right subclavian artery.

A 26-year-old healthy man without any medical history complained of dysphagia. Routine chest radiography showed enlargement of the upper mediastinum at the sternal angle, indicating the presence of an arch anomaly. A barium swallow revealed an indentation of the esophagus on its right aspect. Computed tomographic angiography (CTA) scan showed the presence of a right-sided aortic arch, with the left common carotid artery originating first, right common carotid artery second, followed by the right subclavian artery (RSA) and LSA arising from a Kommerell diverticulum that compressed the esophagus ( Fig. 1 ).

The patient presented with dysphagia because of esophageal compression, so endovascular treatment was contraindicated and the patient underwent open repair.

The patient was taken to the operating room. Selective lung ventilation was used. Cerebrospinal fluid drainage (LiquoGuard, Möller Medical GmbH) was applied to prevent paraplegia.

**Fig. 2:**
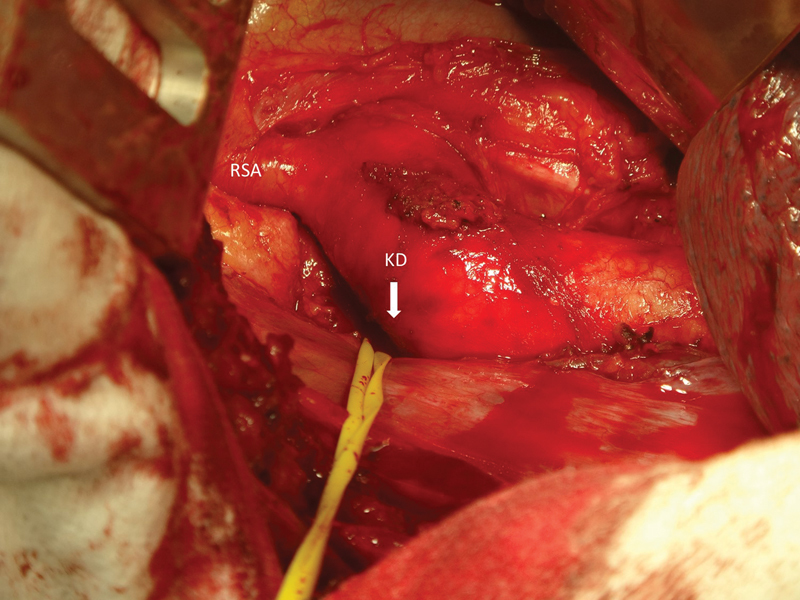
Intraoperative photo. Note the RSA and the KD in the left side of the descending aorta toward the left hemithorax. KD, Kommerell's diverticulum; RSA, right subclavian artery.

A right thoracotomy was performed in the third intercostal space. The distal aortic arch and the RSA were identified and dissected. A careful dissection of the posterior wall of the Kommerell diverticulum adjacent to the vertebral body was undertaken in the left hemithorax until the LSA was of normal diameter. The vessel was surrounded with a silicone tape ( Fig. 2 ). That posterior approach prevented any injuries from the Kommerell dissection adjacent to the esophagus. A strategy of simple side-biting division of the aberrant subclavian at the junction with the aorta was considered unsafe, because of posterior location of the diverticulum (in the left hemithorax), and the necessity to evert the aorta for clamping. Graft replacement with left heart bypass was the chosen technique. Heparin 1 mg/kg was administered. Left heart bypass was established from the right inferior pulmonary vein to the descending thoracic aorta under mild hypothermia (32–34°C). Aortic clamping was done between the right carotid artery and RSA to have a good aortic wall for a safe anastomosis. One vascular clamp was placed distal to the right carotid artery and another in the descending thoracic aorta. Small bulldog clamps were placed on the RSA and the distal LSA. The aorta was transected distal to the RSA and distal to the Kommerell diverticulum insertion. The aorta was opened and an end-to-end anastomosis with a 26 mm Dacron vascular graft was performed between the distal arch and the distal descending thoracic aorta. Because the distal stump of the LSA was far away in the left hemithorax (precluding a safe end-to-end anastomosis), it was transected and oversewn with a 5/0 monofilamentous suture. After releasing the vascular clamp, the stump retracted back to the left and the esophagus was freed from the vascular compression. After protamine administration, pulse oximetry of the left hand showed 100% saturation, so supraclavicular left carotid–subclavian artery bypass was not considered necessary. The postoperative recovery was uneventful and the patient was discharged home on postoperative day 7.

**Fig. 3:**
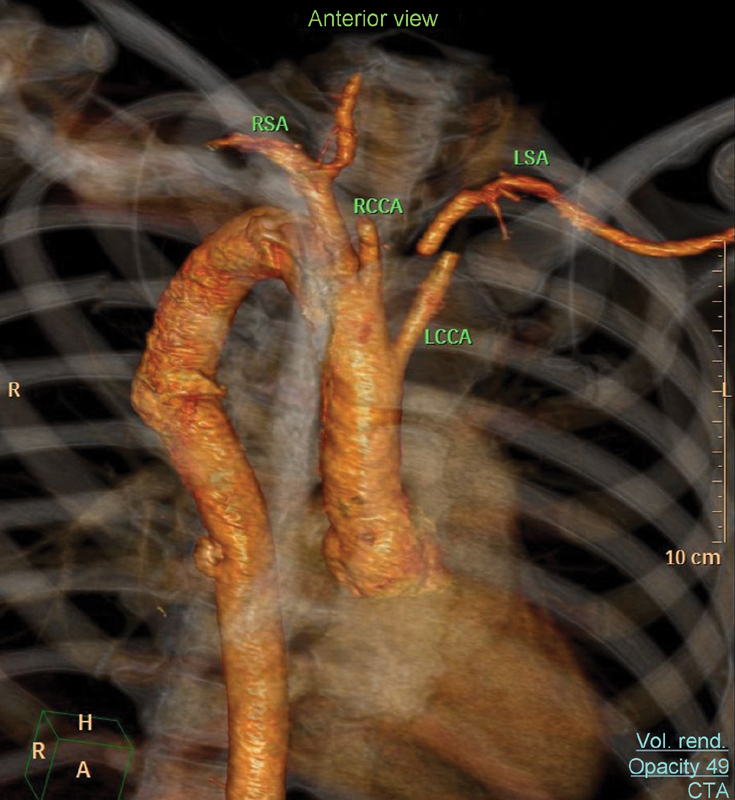
Postoperative computed tomography angiography. Note that the LSA is not connected to the descending aorta and is fulfilled through collaterals. LCCA, left common carotid artery; LSA, left subclavian artery; RCCA, right common carotid artery; RSA, right subclavian artery.

The patient was free of dysphagia at 10 months postoperative without any other symptoms. Follow-up barium swallow was normal and CTA showed complete exclusion of the Kommerell diverticulum and adequate subclavian blood flow ( Fig. 3 ).

## Discussion

Right-sided aortic arch is a rare congenital defect of the aorta and its branches. The frequency of occurrence is 0.05 to 0.1% of the population. 1 Two major variations in right aortic arch can be identified. Type I presents with mirror image of the normal, and type II presents with an aberrant LSA that arises as a fourth branch off the aortic arch, like the present case. 2

David Bayford in 1794 first described an aberrant RSA (ARSA) compressing the esophagus that can lead to clinical symptoms of dysphagia, which, in his words, “may be called lusoria, from Lusus Naturae that gives rise to it.” (The English translation of the Latin term lusus naturae means “freak of nature”). 3 Burckhard F. Kommerell reported in 1936 an aortic diverticulum in a patient who had a left-sided aortic arch and an ARSA. The aneurysmal diverticulum of the descending aorta at the origin of ARSA is called Kommerell diverticulum, which consists of both an aneurysm of the thoracic aorta and an aneurysmal orifice of the aberrant subclavian artery. 4

Surgical indications have not been clearly established because the condition is infrequent. The usual indications for treatment in the adult population are symptoms of tracheal or esophageal compression and, in asymptomatic patients, aneurysmal dilatation (> 5.0 cm) or dissection of the descending thoracic aorta, or the presence of a Kommerell diverticulum > 2 to 3 cm. 5 6 7

This is a serious condition. In review of the literature of aneurysms associated with a right-sided arch reported by Cinà et al, 53% of the 32 cases collected presented with rupture or dissection. 7

Various open and endovascular techniques have been used to treat aberrant LSA in right aortic arch. Endovascular treatment in the presence of esophageal or tracheal compression is not optimal because symptoms may persist after stent exclusion. 6 Open surgical repair is the preferred solution. However, surgical procedures described most often use cardiopulmonary bypass with or without deep hypothermia, and in-hospital mortality reported in the literature for open repair of right-sided aortic arch with Kommerell diverticulum is 5.8 to 18%. 6 7 8 Left subclavian revascularization is recommended; however, in the series of Albacker et al (seven patients) it was not performed, and no patient developed symptoms.

A strategy of simple side-biting division of the aberrant subclavian at the junction with the aorta seems dangerous in the adult because of the thin-walled aorta at that level, wide aneurysmal implantation, and the necessity to evert the aorta for clamping, so we believe as do Kouchoukos and Masetti that the best technique is aortic resection and graft replacement. 9

The present strategy of left heart bypass without LSA reimplantation appears safe. In case of arm symptoms (unlikely), a second stage, extra-anatomic supraclavicular left carotid–left subclavian bypass is an easy option.
